# Update on intraoperative radiotherapy: new challenges and issues

**DOI:** 10.3332/ecancer.2018.793

**Published:** 2018-01-10

**Authors:** Emanuela Esposito, Michael Douek

**Affiliations:** 1 Department of Clinical Medicine and Surgery, University of Naples Federico II, Italy; 2 Division of Cancer Studies, King’s College London, 3rd Floor Bermondsey Wing, Guy’s Hospital, Great Maze Pond, London SE1 9RT, United Kingdom

**Keywords:** breast cancer, intraoperative radiotherapy (IORT)

## Abstract

Intraoperative radiotherapy (IORT) for breast cancer has challenged the standard external beam radiotherapy (EBRT) and has been shown to be non-inferior for treating early breast cancer in the past decade. Several technologies have been tested for IORT and various randomised controlled trials are still ongoing. Different methods of application of IORT have also been evaluated, from early breast cancer to tumour bed boost radiotherapy amongst high risk women. TARGIT-A and ELIOT trials have reported a low incidence of local recurrence and good survival in both arms. Moreover, mortality has been found to be lower amongst women who underwent partial breast radiotherapy compared to those treated with EBRT in a recent meta-analysis. Despite this, IORT has not been introduced in the current clinical practice as yet, and many clinicians do not mention this treatment option to patients awaiting breast cancer surgery. The scientific community does not unanimously support the effectiveness of IORT and still raises concerns about introducing IORT as a standard treatment option for breast cancer. Current evidence demonstrates that IORT is ready for roll-out; it is time to let well-selected and informed patients be offered this treatment option in the current clinical practice.

## Background

Intraoperative radiotherapy (IORT) is the administration of radiation therapy at the time of surgery, accurately defining the target volume of the breast. IORT has gained interest as an alternative to external beam radiation treatment (EBRT) in the past two decades [1]. The rationale for IORT is based on the observation that over 90% of local recurrences after breast conserving surgery (BCS) occur at or near the original operation site [2, 3]. The rate of local recurrence in the remaining breast tissue in other quadrants is 4% [4, 5], which approaches the estimated risk of developing contralateral breast cancer. The milestone study of Holland *et al* [4] has shown that multiple tumour foci occur in up to 60% of mastectomy specimens away from the index quadrant. Trials comparing BCS and mastectomy have demonstrated equivalent survival, suggesting that these small disease foci are not clinically relevant [6].

IORT can be delivered using different techniques, using either low-voltage X-rays or electrons. Two randomised controlled trials, TARGIT-A and ELIOT, have shown, in selected patients, encouraging results in terms of local recurrence and survival.

## IORT for early breast cancer

### ELIOT Trial

ELIOT was a prospective single-centre randomised phase III equivalence trial (**ClinicalTrials.gov Identifier:** NCT01849133). The aim of this trial was to compare a 21 Gy single-dose IOERT delivered using the ELIOT technique to conventional whole breast EBRT [7]. The pre-specified equivalence margin was 7.5% (90% of statistical power at 5% significance) and a non-inferiority margin of 4.5%. A total of 1,305 women were randomised, aged between 48 and 75 years, with clinically invasive T1–T2 ≤ 2.5 cm breast cancers suitable for BCS. Detailed inclusion criteria are listed in Table 1. After five years’ follow-up, the ELIOT trial showed a 4.4% of local recurrence rate (LRR) amongst patients who underwent BCS and IORT. The Italian retrospective analysis of the ELIOT technique showed that the five-year LRR increased as patients moved from ASTRO ‘suitable’ to ‘cautionary’ to ‘unsuitable’ groups (1.5%, 4.4% and 8.8%, respectively). Similarly the five-year LRR for ‘low risk’, ‘intermediate risk’ and ‘high risk’ groups according to the GEC-ESTRO guidelines was 1.9%, 7.4% and 7.7%, respectively [8–11].

### TARGIT-A Trial

The TARGIT-A was a prospective multicentre phase III trial that enrolled 3,451 patients over 45-years old with clinically T1–T2 ≤ 3.5 cm, N0–1 invasive tumours (**ClinicalTrials.gov Identifier:** NCT00983684). Inclusion criteria are shown in Table 2. The device used to deliver IORT was Intrabeam® by Carl Zeiss, which uses low-energy X-rays (50 kV). Randomisation occurred either before surgery (prepathology cohort; IORT concurrent with BCS) or after surgery (postpathology cohort; IORT given subsequently as a second surgical procedure). Approximately 30% of the patients received IORT postpathology during a second surgical procedure, and were randomised within 30 days after the original operation. A ‘risk-adapted approach’ was used for IORT, meaning that if the final pathology showed pre-specified adverse features, EBRT was administered after surgery. The estimated recurrence rate was (3.3%; 95% confidence interval [CI] 2.1–5.1%) after IORT compared to (1.3%; 95% CI 0.7–2.5%; p = 0.042) after EBRT at 3.8 years median follow-up (35% of patients reached five years follow-up). The pre-specified non-inferiority margin was 2.5% (80% of statistical power at 5% significance level), based on an estimated LRR of 6% with EBRT. The 2.5% non-inferiority margin was met in this trial both for these overall data and in the prepathology cohort (IORT 2.1 (1.2–4.2%) *versus* EBRT 1.1 (0.5–2.5%); P =0.31), but not in the postpathology cohort (5.4 (3.0–9.7%) *versus* 1.7 (0.6–4.9%), respectively; P = 0.069).

### TARGIT-US trial

TARGIT-US is a phase IV registry trial launched in 2012 by Michael Alvarado and colleagues in the USA. This study proposes to investigate the efficacy and toxicity of IORT after BCS, with or without EBRT as indicated by pathologic risk factors, in women with early stage breast cancer (**ClinicalTrials.gov Identifier:** NCT01570998). The primary endpoint is in-breast local failure. The secondary endpoints are toxicity and morbidity, relapse-free survival and overall survival. The estimated sample size is 750 patients. Patients selected for BCS, who are considered to have a low risk of local recurrence, are eligible for the registry trial once given their informed consent. Inclusion criteria are shown in Table 3. Patients receive IORT as a single fraction over 15–40 minutes at the time of lumpectomy. The technique and doses used are the same as the TARGIT-A trial, but the accrual is only open to IORT at the time of the initial lumpectomy and there is no postpathology randomisation. The trial is still ongoing and is recruiting patients – it is expected to achieve complete accrual in 2017.

### TARGIT – Retrospective

In September 2016, Valente *et al* [12] published the first analysis of a multi-institutional retrospective registry using Intrabeam® in North America. 19 institutions from the United States and Canada retrospectively entered data on the use of IORT. 935 women underwent lumpectomy and IORT either concurrent to surgery (prepathology stratum) or after reopening the wound or even like a programmed boost from 2007 to 2013. The registry has shown that the number of women treated with Intrabeam® has increased over the years (p = 0.005). The median age was 66.8 years. 90% of the patients selected presented with T1 tumours with oestrogen receptors positive. 83% of tumours were grade 1 or 2, 79% were in the prepathology stratum, 7% in the postpathology stratum, whereas 14% received IORT as a boost. At a median of 23.3 months of follow-up, 2.3% of in-breast true recurrences were observed. In a per-stratum analysis, 2.4% of recurrences were found in the prepathology cohort, whilst in the postpathology cohort the recurrence rate was 6.6%. The TARGIT-R study confirmed that IORT performed concurrently at the time of lumpectomy is the preferred approach.

## IORT as a boost after breast conserving surgery

### TARGIT-B Trial

In February 2013, the TARGIT-B trial was launched (**ClinicalTrials.gov Identifier:** NCT01792726) by University College London. TARGIT-B aims to compare IORT boost with EBRT boost in early breast cancer. TARGIT-B is a multicentre randomised controlled trial designed to test the hypothesis that tumour bed boost delivered by IORT is superior to the standard external beam tumour bed boost administered in five fractions over five days. The trial is still recruiting patients. The device used to deliver IORT is Intrabeam® by Carl Zeiss. Eligible patients are those awaiting BCS who are found to possess one or more risk factors for local recurrence at core biopsy. The accrual goal is 1,796 patients. Table 4 reports inclusion criteria for TARGIT-B trial. Patients are randomised into two groups, namely, the boost group and the EBRT group. In the boost group, 20 Gy IORT boost is delivered to the tumour bed after tumour resection over 20–35 minutes, whereas a standard external beam tumour bed boost is administered along with EBRT to the EBRT group. All patients enrolled within this trial receive postoperative EBRT and adjuvant treatments according to the pathology final report. The primary outcome is local tumour control. The secondary outcomes are site of relapse; five years’ relapse-free survival; overall survival; local toxicity and morbidity; and quality of life.

## IORT in elderly patients

### TARGIT-E Trial

TARGIT-E(lderly) is a multicentre single-arm prospective phase II study of IORT in elderly patients with small breast cancer (**ClinicalTrials.gov Identifier:** NCT01299987) [13]. The TARGIT-E trial is based on the TARGIT-A trial and IORT is administered using Intrabeam® (Carl Zeiss). The aim is to investigate the efficacy of IORT amongst elderly patients with small breast tumours. Table 5 shows inclusion criteria for the TARGIT-E trial. EBRT is administered only if the final pathology demonstrates additional risk factors for local recurrence. The TARGIT-E trial is based on the rationale that local recurrence among women aged 70 years and older is about 4% and drops to 1% when radiotherapy plus tamoxifen is given [14]. Launched in 2011 by Universitätsmedizin Mannheim, TARGIT-E has recruited 538 patients, although the estimated accrual goal was 265. On November 2017, final data collection for the primary outcome measure is expected to be released [15]. The primary outcome is local relapse rate. Secondary outcomes include cancer-specific and overall survival, rate of contralateral breast cancer, quality of life and cosmetic outcome. The expected local relapse rates are 0.5%, 1.0% and 1.5% after 2.5, 5.0 and 7.5 years, respectively.

## Latest ongoing trials

### Prospective cohort study

TARGIT-C (Consolidation) trial is a prospective phase IV trial first launched on October 2014 (**ClinicalTrials.gov Identifier:** NCT02290782) by Universitätsmedizin Mannheim. This prospective, multicentre single-arm phase IV study is based on the protocol of the international TARGIT-A and Intrabeam® is used to deliver IORT. The endpoints are the same as those for the TARGIT-E trial, namely, local relapse rate, cancer-specific and overall survival, rate of contralateral breast cancer, quality of life and cosmetic outcome. The expected local relapse rates are 0.825–1.375% after 3–5 years, respectively. The estimated sample size is 387 patients. Inclusion criteria are shown in Table 6. This trial is currently recruiting participants. The rationale for this trial is based on the observation that the efficacy of radiation of the tumour bed only in a selected group can be non-inferior to whole EBRT [16–18]. TARGIT-C trial aims to confirm the efficacy of a single dose of IORT in a well-selected group of patients with small breast cancer and absence of risk factors, as has been shown in the TARGIT-A trial by Vaidya *et al* [17, 18].

### IORT delivered with Xoft® Axxent® eBx™

The purpose of the phase IV Xoft® Axxent® eBx™ IORT trial is to assess the safety and efficacy of the Xoft® Axxent® eBx™ System when used for single-fraction IORT in early stage breast cancer (**ClinicalTrials.gov Identifier:** NCT01644669). Xoft® is a balloon catheter born for brachytherapy with single-entry, which can be inserted into the tumour cavity by the surgeon at the time of surgery or after operation. The Xoft® Axxent® eBx™ System has been used to treat early breast cancer with a multifraction accelerated partial breast irradiation (APBI) technique on an outpatient basis as a part of two multicenter studies [19]. The disadvantage of brachytherapy balloons is that the radiation treatment is not concluded at the time of the operation, but once the device has been placed; radiation is delivered in ten fractions twice a day over five consecutive days. The Xoft® Axxent® eBx™ System balloon, which uses low-energy X-rays (50 kV), is the only balloon device now being tested for single-dose IORT [20]. Results from this phase IV trial study aimed at assessing clinical efficacy and safety are still awaited. The accrual goal of this trial is 1,200 patients and the first patient was recruited in 2012. The primary outcome is ipsilateral breast tumour recurrence at five years’ follow-up. The secondary outcomes are regional breast tumour recurrence; disease-free survival and overall survival as well as cosmetic outcome, at five and ten years. Eligibility criteria for enrolment are shown in Table 7.

## Effects on mortality of IORT in early breast cancer

A meta-analysis of randomised trials by Vaidya *et al* [21] analysed mortality differences in randomised trials of partial-breast irradiation (PBI). Nine randomised trials of PBI versus whole breast external beam radiation in invasive breast cancer were identified, although PBI was delivered with different techniques. For the TARGIT-A trial, data from 1,222 patients were available for the meta-analysis as only this subgroup of patients reached five years’ follow-up. Five-year outcomes were available for non-breast cancer mortality in five trials and for breast cancer mortality in four trials. The overall survival was 94.6% for PBI versus 91.85% for EBRT. There was no difference in the proportion of patients dying of breast cancer (difference, 0.000% [95% CI 0.7–þ 0.7]). Non-breast cancer mortality with PBI was lower than with whole EBRT (difference, 1.1% [95% CI 2.1–0.2%]). Total mortality with PBI was also lower than with whole EBRT (difference, 1.3% [95% CI 2.5–0.0%]). The authors concluded that use of PBI instead of whole EBRT results in a lower five-year non-breast cancer and overall mortality. Moreover, the authors stated that patients should be informed about these data when breast conserving therapy is proposed.

## Discussion and conclusions

The use of IORT as an alternative to EBRT in selected groups of patients has been a fundamental change in approaching breast cancer therapy. The North American TARGIT-R (Retrospective) Registry has shown low recurrence rate and low complication after lumpectomy and IORT at a median follow-up of 23.3 months [12]. Two randomised controlled trials, TARGIT-A and ELIOT, have shown that IORT is non-inferior to EBRT in terms of LRR when delivered to patients with early breast cancers and specific tumour characteristics. Based on the TARGIT-A findings, the TARGIT-US trial represents a pragmatic registry designed to follow the outcomes of IORT around the USA. The German TARGIT-C trial is still ongoing and aims to consolidate outcomes from TARGIT-A by using the same technique. The German TARGIT-E trial was launched to demonstrate that elderly patients, who are often undertreated as they often do not comply with standard 3–6 weeks of EBRT, should be treated at the time of surgery with IORT when they present with a small breast cancer. The ongoing TARGIT-B trial is evaluated IORT boost amongst young and high-risk patients, as the tumour bed boost is often missed (20–90%) due to the tissue displacement and the frequent lack of cavity clips during oncoplastic BCS [22, 23]. Moreover, new technologies are now tested to deliver radiation therapy entirely at the time of surgery, such as Xoft® Axxent® eBx™ System balloon, born and launched in the market for brachytherapy to deliver multifraction radiotherapy for five consecutive days. The Xoft® Axxent® eBx™ System balloon is now used for single-fraction IORT and its efficacy is still being evaluated in the USA. With regard to mortality associated with radiation treatment, the recent meta-analysis published by Vaidya and colleagues demonstrated benefits from PBI compared to whole EBRT, although there was heterogeneity between the trials for many of the outcomes. It still remains unclear how many years of follow-up are needed to obtain solid information on non-breast cancer-related death as historically 10–15 years should be awaited before this data can be confirmed.

To conclude, current evidence suggests that it is time for a paradigm shift to inform patients about IORT and offer selected patients the option of IORT during BCS for cancer.

## Conflicts of interest

The authors have no disclosures to make concerning financial and personal relationships with other people or organisations that could inappropriately influence their work.

## Figures and Tables

**Table 1.** Inclusion criteria for ELIOT trial.

Age ≥ 48 and < 75
Unifocal invasive breast carcinoma ≤2.5 cm.
No previous therapy (biopsy included) for breast cancer in other institutions.
Informed consent

**Table 2.** Inclusion criteria for TARGIT-A trial.

Age ≥45 years
Invasive breast cancers other than lobular
T1 and T2 (<3.5 cm), N0-1, M0
Suitable for breast conserving surgery Available for follow-up for at least ten years
Previously diagnosed and treated contralateral breast cancer

**Table 3.** Inclusion criteria for TARGIT-US trial.

Age ≥45 years
Invasive breast cancers other than lobular
T1 and T2 (<3.5 cm), N0-1, M0
ECOG performance status of 0–3
Informed consent

**ECOG** = Eastern Cooperative Oncology Group

**Table 4.** Inclusion criteria for TARGIT-B trial.

Age <45 years
Lobular carcinoma
Post neo-adjuvant systemic therapy
Age ≥45 years with G3 tumour ER negative, PR negative Positive margins Lymphovascular invasion Gross nodal involvement (not micrometastasis) More than one tumour in the breast but still suitable for BCS Patients with either HER2 positive or HER2 negative

**ER**= oestrogen receptor

**PR**= progesterone receptor

**BCS**= breast conserving surgery

**Table 5.** Inclusion criteria for TARGIT-E trial.

Age ≥70 years
Unilateral and unifocal breast cancer
cT1c N0 M0
Invasive ductal carcinoma
Absence of lymphovascular invasion
Compliance and informed consent

**Table 6.** Inclusion criteria for TARGIT-C trial.

Age ≥50 years
Invasive-ductal breast cancer
Hormone receptor positive
Tumour size ≤3.5 cm cN0 cM0
≥50 years of age
Compliance and informed consent

**Table 7.** Inclusion criteria for Xoft® Axxent® eBx™ IORT trial.

Age ≥40 years
Invasive ductal carcinoma or ductal carcinoma in situ of the breast
Tumours smaller than 3.0 cm [Tis, T1 or T2 (<3 cm), N0, M0]
Bilateral breast cancer if both cancers me*et al*l of the inclusion
Informed consent
